# Life events, emotional responsiveness, and the functional prognosis of patients with rheumatoid arthritis

**DOI:** 10.1186/s13030-015-0043-3

**Published:** 2015-06-23

**Authors:** Jun Nagano, Nobuyuki Sudo, Shohei Nagaoka, Masao Yukioka, Masakazu Kondo

**Affiliations:** Faculty of Arts and Science, Kyushu University, 6-1 Kasuga Park, Kasuga, Fukuoka 816-8580 Japan; Department of Psychosomatic Medicine, Kyushu University Graduate School of Medical Sciences, 3-1-1 Maidashi, Higashi-ku, Fukuoka 812-8582 Japan; Yokohama Minami Kyousai Hospital, 1-21-1 Mutsu-ura Higashi Kanazawa-ku, Yokohama, 236-0037 Japan; Yukioka Hospita, 2-2-3 Ukida, Kita-ku, Osaka 530-0021 Japan; Kondo Rheumatism and Orthopedics Clinic, 3-10-11 Tenjin, Chuo-ku, Fukuoka 810-0000 Japan

**Keywords:** Arthritis, Rheumatoid, Stress, Psychological, Personality, Emotions, Function, Prospective studies

## Abstract

**Background:**

Stressors may differently affect human physiological systems according to the host properties relevant to psycho-behavioral processes that the stressors invoke. In a Japanese multicenter cohort study of patients with rheumatoid arthritis (RA), we examined if major life events differently contribute to the patients’ functional prognosis according to their ability to identify emotions as manifest feelings when encountering the events (emotional responsiveness).

**Methods:**

460 patients with RA completed a self-administered baseline questionnaire about psychosocial factors including emotional responsiveness. Two years later, they checked on a list of positive/negative personal events that happened during the two-year study period. Rheumatologists evaluated their functional status at baseline and follow-up using the ACR classification system.

**Results:**

In a multiple logistic regression model that included baseline demographic, disease activity/severity-related, therapeutic, and socioeconomic factors as covariates, none of the counts of positive, negative, or all life events was associated with the functional status at follow-up. In the subgroup with poor emotional responsiveness, however, these life event counts were all associated with a poorer functional prognosis (odds ratio of ACR class 3–4 vs. 1–2 associated with one increment in the all life-event count = 2.39, 95 % confidence interval = 1.27-4.48, *p* = .007), while no such relationship was evident for the rest of the patients.

**Conclusions:**

Major life events, whether positive or negative in nature, may have an impact on the disease course of patients with RA when the patient has poor emotional responsiveness to the event(s).

## Background

Psychological stress (stress) is determined not only by external stressors, but also by interactions with internal conditions, i.e., emotional, cognitive, and behavioral response properties [1]. Thus, it is important to take individual response properties into consideration when addressing the effects of external events that place stress on the organism. Major life-event type stressors alone have not been observed to precede the onset of rheumatoid arthritis (RA) [2], an autoimmune disease characterized by chronic systemic inflammation that mainly affects joints leading to a loss of physical functioning [3], although the psychosomatic aspects of RA have been considered for decades [4, 5]. Moreover, life events have been suggested to be associated with a decrease in the disease activity of RA [6]. As regards individual properties (reaction to stressors), however, patients with RA who are easily moved to tears as a response to psychological stress have been reported to show a better response to treatment and a better general prognosis than those who did not show such an emotional response [7]. In addition, we recently reported that “rational and antiemotional” behavior (antiemotionality), characterized by an extreme tendency to suppress emotional behaviors and to rationalize negative experiences in conflicting interpersonal situations, is associated with a poor functional prognosis for patients with RA [8].

In order to invoke such cognitive and behavioral responses as expression/suppression of emotions, however, life events must arouse emotions that patients are aware of and that they identify as feelings, i.e., subjective and conscious experiences [1, 9, 10]. Persons who lack strong, positive or negative emotional experiences in life may have diminished ability to detect and identify emotions or have relevant processes that are strongly suppressed. When emotions are inappropriately processed, the limbic system, the center of emotions, might interfere with the homeostasis of the organism, including the immune system, through the hypothalamus-pituitary-adrenal (HPA) axis and the hypothalamus-autonomic nervous system (ANS), thereby affecting the functional prognosis of patients with RA [6, 11, 12]. We tested these hypotheses by calculating the self-reported major life events that occurred within a given period, two years, with a lifelong lack of emotional experiences as an index for poor detection/identification of emotions, antiemotionality as a poor coping behavior, and the transition of physical functional status as an organic change, on the basis of data from a cohort study of Japanese RA patients.

## Methods

### Subjects

**Fig. 1:**
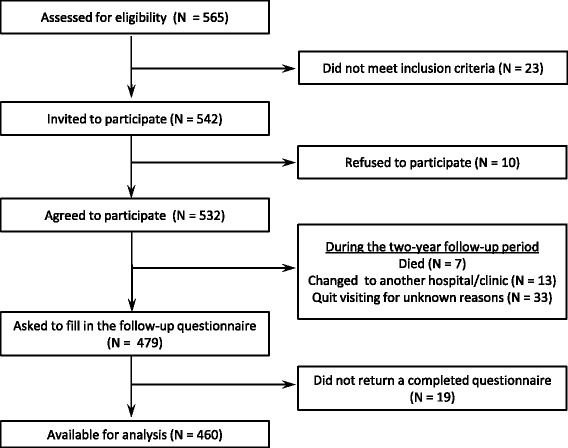
Flow diagram

The subjects were patients who participated in the Assessment and Improvement of the System for Interdisciplinary Medical Services for RA (AISIMS) cohort study [13] and whose follow-up data two years after the baseline survey was available. The AISIMS cohort study is a multi-center survey of Japanese patients with RA that started in 2000 with support from the Ministry of Health and Welfare of Japan. Twelve hospitals/clinics located across Japan participated in the study program, of which eight cooperated in the follow-up survey. A series of 532 eligible patients with RA who were regularly visiting one of the eight hospitals/clinics and who met the following criteria completed a self-administered questionnaire for the baseline survey in 2000. The survey inquired about a variety of factors, including activity of daily living, quality of life, lifestyle, and psychosocial factors (major life events, stress/personality, etc.). Eligibility criteria were age between 20 and 79 years, the ability to answer a self-administered questionnaire without assistance, and functional status of class III or lower according to the American College of Rheumatology (ACR) (see below) [14]. A total of 23 rheumatologists responsible for the patients completed a baseline clinical data sheet that included information about the disease status, such as the progression of arthritis, functional status, extra-articular complications, and medical treatment. The same rheumatologists again completed the clinical data sheet for each of the 479 patients who continued visiting to the time of the follow-up survey, which included a questionnaire with items selected from the baseline questionnaire. The 460 cooperating patients (406 women, mean age 56.1 years) mailed their completed questionnaires to a central office (see Fig. 1). The rheumatologists who completed the clinical data sheets were blinded to the patients’ answers for both the baseline and follow-up questionnaires. A more detailed description of the subjects has been reported elsewhere [8].

### Measurements

The rheumatologists assessed their patients’ functional status based on the criteria for the classification of functional status of RA patients defined by ACR (ACR class), which classifies the patients into one of four classes as follows: Class I, completely able to perform usual activities of daily living (self-care, vocational, and avocational); Class II, able to perform usual self-care and vocational activities, but limited in avocational activities; Class III, able to perform usual self-care activities, but limited in vocational and avocational activities; and Class IV, limited in ability to perform usual self-care, vocational, and avocational activities [14]. They also assessed joint damage based on the classification by Steinbrocker et al. (joint stage: Stage I, Early; Stage II, Moderate; Stage III, Severe; and Stage IV, Terminal) [15]; specified afflicted joints defined as those with either tenderness, swelling, or deformity; and listed extra-articular complications using the following options: cervical myelopathy, cardiac/pericardial manifestations, pulmonary/pleural manifestations, ocular manifestations, peripheral nervous manifestations, hematological manifestations, and others.

Major life events that occurred in the two-year follow-up period were assessed using a self-administered questionnaire that was developed specifically for the ASIMS cohort study. It consists of an instruction sentence, “please answer if any of the following eight major life events happened to you in the past two years”: 1. Got married; 2. Went through bereavement or parted with an important person; 3. I or my partner got a new job; 4. I or my partner lost a job (excluding retirement); 5. Financially became much better off than before; 6. Financially got into a serious situation; 7. A major family problem was resolved; and 8. A major family problem occurred. Items 1, 3, 5, and 7 are positive events, and items 2, 4, 6, and 8 are negative events. These items were prepared referring to reports that graded major life events according to the possible magnitude at which the events would impact life [16]; the grading is very similar between the original survey in the U.S.A. and replication studies done in Japan [17, 18]. The subjects ticked either “happened” or “did not happen” for each item. The same questionnaire, with one difference in that the instruction sentence referred to life events that happened in the previous year, was also included in the baseline survey to attempt to cross-validate it with an instrument that measures emotional responsiveness to major life events (see below). This issue is discussed in the Discussion section of this paper.

The psycho-behavioral properties detection and identification of emotions and coping behaviors were assessed using the “Stress Inventory (SI)” [19, 20]. The SI is a 45-item self-administered questionnaire that was developed to assess response styles to stressors principally related to an interpersonal relationship or to chronic stress posed by the response style. Of the 12 scales constituting the SI, the “lack of emotional experiences (LEE)” scale attempts to assess poor emotional responsiveness to major life events, i.e., diminished ability to detect and identify strong emotions as manifest feelings when a person encounters positive and negative major life events. This scale consists of four questions, such as “in your whole life, have you experienced outrage about something?” and “in your whole life, have you experienced jumping for joy about something?” (Cronbach α = 0.60). The “rationalizing conflicts/frustrations (RCF)” scale was developed to represent antiemotionality. It measures an extreme tendency to rationalize one’s interpersonal situations accompanied by conflicts or frustrations and it consists of five questions, such as “do you under all circumstances try to control your reasoning and avoid, as much as possible, being emotional?” (Cronbach α = 0.78). The answers receive a 1 to 6 rating, where 1 and 6 respectively correspond to “yes” and “no”, and the scores are averaged for the scale score, thus a higher score represents a higher tendency. While the concept of antiemotionality represents a behavioral response style in daily situations that distinctly arouse conflicting or frustrating emotions, the core concept of poor emotional responsiveness represents a more biological, rather than behavioral, response style when encountering major life events [19]. In a psychometrical validation study, for example, while RCF was moderately correlated with anger traits, LEE was only weakly correlated, if any [20]. The RCF and LEE scores in the present study were statistically independent (Spearman’s rank correlation coefficient = −0.03).

### Analysis

The ACR class at follow-up was dichotomized (poorer function, Class III or IV vs. better function, Class I or II) for use as the outcome variable representing the functional prognosis of the patients with RA. “Happened” items in the life event questionnaire were counted separately for positive (0–4) and negative events (0–4), and also aggregated for all events (0–8). Thus for example, if a patient ticked two positive event “happened” boxes and one negative event “happened” boxes, the life event counts for the patient would be 2, 1, and 3 for positive, negative, and all events, respectively. The associations between these life event counts and the functional prognosis were examined using a multiple logistic regression model that included one of these life event counts as an independent variable, the dichotomized ACR class at follow-up (better function = 0, poorer function = 1) as the dependent variable, and baseline ACR class (Class I, II, III, IV = 1, 2, 3, 4, respectively) as a covariate. The models also included known or potential confounding factors relevant to disease progression or activity: joint stage (Stage I, II, III, IV = 1, 2, 3, 4, respectively), afflicted joints count (1–45), number of extra-articular complications (0–5), C-reactive protein with log-transformation; medical treatments: methotrexate, corticosteroids, other DMARDs (yes = 1, no = 0); and socioeconomic status: education level (junior high school = 1, high school = 2, junior college = 3, college = 4). These analyses were then applied to the subgroups divided based on either the degree of the LEE score or that of the RCF score, using the median of each score as the cut-point. High (> = 2.5 points) and low (<2.5 points) LEE scores represent high and low degrees of poor emotional responsiveness; and high (> = 4.4 points) and low (<4.4 points) RCF scores represent high and low degrees of antiemotionality. The reported *p*-values are two-sided, and values < .05 were considered statistically significant. All analyses were done using SAS v. 9.2.

## Results

Table 1 shows the baseline characteristics of the patients according to their level of emotional responsiveness. Poorer emotional responsiveness was associated with male sex, older age, lower education level, more advanced joint stage, higher CRP, and lower corticosteroid use. Greater antiemotionality was associated with female sex, older age, and greater number of afflicted joints: The corresponding table has been presented elsewhere [8]. Table 2 shows the number of subjects who reported having experienced a life event during the two-year observation period. The most frequent event was a bereavement/parting with an important person, followed by a financial crisis, with over 15 % of the patients having experienced one of these negative events. When the events were totaled, the number of patients who reported one event and two or more events were respectively 71 (15.5 %) and 23 (5.0 %) for positive events, 120 (26.1 %) and 58 (12.6 %) for negative events, and 126 (27.5 %) and 93 (20.3 %) for all events.

**Table 1:** Baseline association of poor emotional responsiveness with the demographic and clinical characteristics of patients with rheumatoid arthritis

	Poor emotional responsiveness^a^		*p* ^b^
	Low	High	
	*N* = 252	*N* = 208	
Female	91.7	84.1	.013
Age, yrs, mean (SD)	54.3 (9.7)	58.4 (9.0)	<.001
Education, college or higher	24.2	16.4	.038
Duration, yrs, mean (SD)	11.6 (9.7)	11.1 (9.3)	.58
ACR class > =3	13.5	13.5	.99
Joint stage > =3	62.7	71.6	.043
Afflicted joints count, mean (SD)	9.4 (8.2)	10.6 (9.4)	.15
No. extra-articular complications > =1	22.6	25.0	.55
CRP, mg/dl, mean (SD)	1.42 (1.82)	1.88 (2.40)	.019
Methotrexate use	42.1	42.3	.96
Corticosteroids use	50.4	37.5	.006
Other DMARDs use	55.2	51.4	.43

Values are % unless otherwise stated. ACR: American College of Rheumatology, CRP: C-reactive protein, DMARDs: disease modifying anti-rheumatic drugs. ^a^The SI “lack of emotional experiences” score (19,20): low score < 2.5 points, high score > =2.5 points. ^b^Based on Spearman’s rank correlation

**Table 2:** Positive and negative life events reported to have occurred in the lives of patients with rheumatoid arthritis during the two-year study period

	Number^a^	Percent^b^
Positive events		
1.^c^ Got married.	10	2.2
3. I or my partner got a new job.	28	6.1
5. Financially became much better off than before.	41	8.9
7. A major problem within my family was resolved.	28	6.1
Negative events		
2. Bereavement or parting from an important person.	73	15.9
4. I or my partner lost a job (excluding retirement).	30	6.5
6. Financially got into a serious situation.	71	15.4
8. A major problem came about in my family.	53	11.5

^a^Number of patients who ticked the “happened” box that corresponds to the event, and ^b^proportion [number/total (*N* = 460)]. ^c^Question number

The distribution of ACR class changed from baseline to the follow-up as follows: Class 1, 76 (16.5 %) to 65 (14.2 %); Class 2, 322 (70.0 %) to 327 (71.2 %); Class 3, 62 (13.5 %) to 58 (12.6 %); and Class 4, 0 (0 %) to 9 (2.0 %). The global functional status had improved for 56 (12.2 %) patients, was unchanged for 327 (71.2 %), and had deteriorated for 76 (16.6 %) over the two-year study period. In addition to the ACR class at baseline, the afflicted joints count and steroid use were significantly associated and extra-articular complication was marginally significantly associated with the functional prognosis in a multiple logistic regression model that included the baseline characteristics as independent variables (data not shown). In the multiple logistic regression model that included these baseline factors as covariates, emotional responsiveness was not associated with the functional prognosis (OR associated with one-point increment in the LEE score = 0.97, 95 % CI = 0.71-1.33, *p* = .83). The positive (OR associated with one-event increment = 1.20, 95 % CI = 0.68-2.13, *p* = .53), negative (OR = 1.25, CI = 0.81-1.94, *p* = .32), and all life event counts (OR = 1.31, CI = 0.88-1.94, *p* = .18) were not significantly associated with the functional prognosis in the analysis of all patients.

**Fig. 2:**
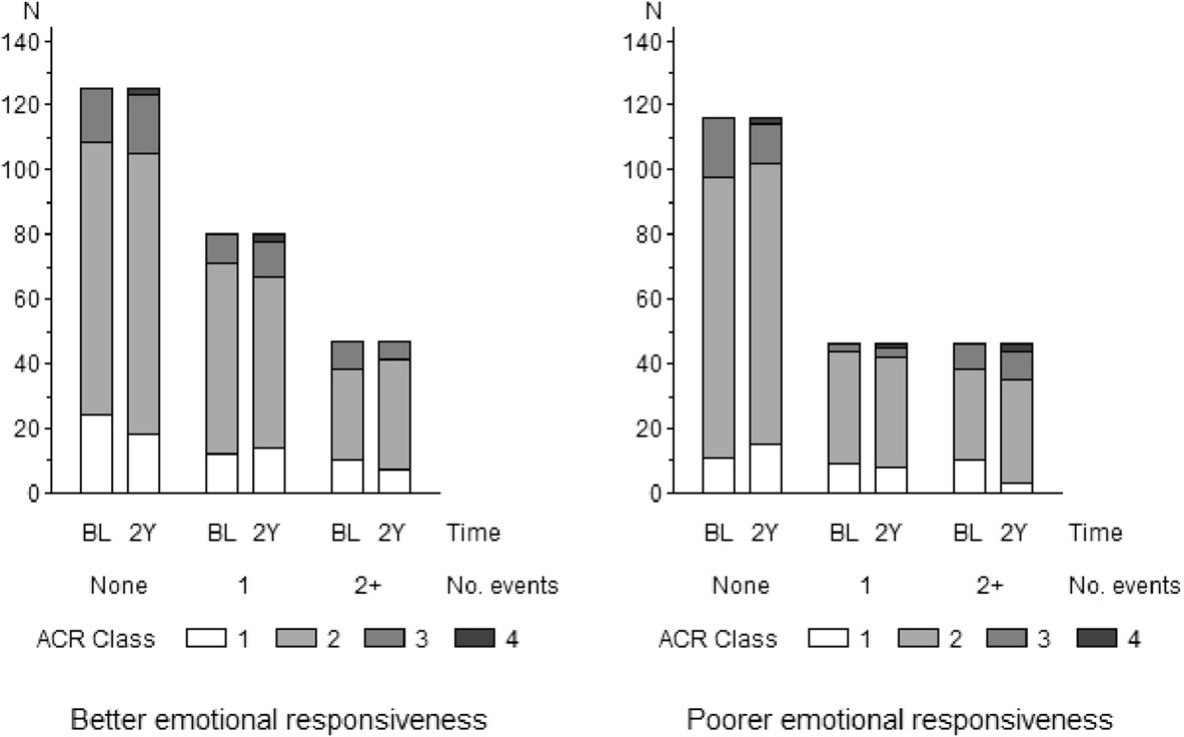
The association of the number of major life events experienced by patients with rheumatoid arthritis in the two-year study period and the transition of their physical functional status according to their degree of emotional responsiveness. N, number of subjects; BL, at the time of the baseline survey; 2Y, two years after the baseline survey. No. events: the number of life events reported to have happened in the two-year study period. ACR: American College of Rheumatology. Better and poorer emotional responsiveness are represented by low (<2.5 points) and high (2.5+ points) scores for the lack of emotional experiences scale from the Stress Inventory (19,20)

As shown in Table 3, however, when the patients were divided into two subgroups by the degree of emotional responsiveness (LEE score), the associations of life events were different. For the patients whose emotional responsiveness was not poor the associations were null or inverse, if any, while for those with poor emotional responsiveness positive, negative, and all events were all significantly associated with poorer functional status at follow-up. Thus, for patients with poor emotional responsiveness, experiencing a major life event was associated with a two or greater fold chance of having a poorer functional status in the subsequent two years. Such a difference in the events/prognosis associations was not evident when the data was stratified by the dichotomized level of antiemotionality (RCF score) (Table 3). Figure 2 shows the transition of ACR class associated with the number of major life events in the study period, according to the degree of emotional responsiveness. In the subgroup with poor emotional responsiveness (right), the percentage of patients whose functional class deteriorated in the study period tended to be higher as the number of life events increased: 10.3 % (12 of 116), 17.4 % (8 of 46), and 23.9 % (11 of 46) for those who reported none, one, and two or more events, respectively. In the subgroup with better emotional responsiveness (left), however, such tendency was not evident, with the corresponding percentages 20.8 % (26 of 125), 15.0 % (12 of 80), and 14.9 % (7 of 47), respectively.

**Table 3:** Multiple logistic regression analysis of the association between life events that occurred during the two-year study period and the functional prognosis of patients with rheumatoid arthritis, stratified by dichotomized levels of the baseline behavioral patterns

Life events	Baseline behavioral pattern											
	Poor emotional responsiveness						Antiemotionality					
	(LEE score^a^)						(RCF score^b^)					
	Low			High			Low			High		
	*N* = 252			*N* = 208			*N* = 217			*N* = 242		
	OR	(95 % CI)^c^	*p*	OR	(95 % CI)	*p*	OR	(95 % CI)	*p*	OR	(95 % CI)	*p*
Positive events	0.60	(0.23-1.53)	.28	2.73	(1.13-6.58)	.026	0.82	(0.27-2.48)	.73	1.46	(0.69-3.11)	.32
Negative events	0.82	(0.42-1.63)	.58	1.99	(1.03-3.86)	.041	1.87	(0.93-3.75)	.08	0.81	(0.42-1.56)	.53
All events	0.77	(0.42-1.40)	.39	2.39	(1.27-4.48)	.007	1.59	(0.83-3.04)	.16	1.11	(0.64-1.94)	.71

OR: odds ratio. CI: confidence interval. ACR: American College of Rheumatology. DMARDs: disease modifying anti-rheumatic drugs. ^a^The lack of emotional experiences (LEE) score of the Stress Inventory (19,20): low score < 2.5 points (median), high score > = 2.5 points. ^b^The rationalizing conflicts/frustrations (RCF) score of the Stress Inventory: low score < 4.4 points (median), high score > = 4.4 points. ^c^OR of poorer (ACR class at follow-up > = 3) vs. better (ACR class at follow-up < =2) functional prognosis associated with one-point increment in the number of life events experienced in the two-year study period, controlled for age, sex, education level, and baseline measurements including ACR class, C-reactive protein, extra-articular complications, methotrexate, corticosteroids, and other DMARDs

## Discussion

This Japanese, multicenter, cohort study of patients with RA found that a lack of experiences accompanied by strong feelings caused by positive and negative life events was associated with a poor functional prognosis. This is, to the authors’ knowledge, the first epidemiological study to report the possibility that life-event type stressors impact the disease course of RA patients according to their ability to detect and identify their emotions.

The death of a child as a major life event has been reported to not increase the risk of onset (new admission) of RA of the parents [2]. This is in line with the present finding that life events were not associated with the functional prognosis of these RA patients when the data is taken as a whole. However, when the data is limited to patients who were thought to have poor emotional responsiveness as an antecedent condition, life events, irrespective of their positive or negative nature, were associated with their functional prognosis.

The four items of the LEE scale asked the patients if they had *“not at all”* experienced an incident accompanied by a strong positive or negative emotional response. Of the patients, 77 basically agreed to these questions (3.5 points or higher on average), but 18 %, 38 %, and 43 % reported having experienced one or more positive, negative, or any life event, respectively, during the year previous to the baseline survey. Moreover, these proportions were similar for the other 383 patients (20 %, 35 %, and 46 %, respectively). Thus, it would be more natural to think that even major events have minimally aroused emotional responses that those who relatively favored the LEE questions are manifestly aware of, rather than that no major event had actually happened in their life. When events arouse well-identified feelings, a person would initiate coping behaviors, thereby alleviating the events’ potential health effects [10]. On the other hand, external stimuli, when they were not sensed, may affect the immune system through the HPA axis and the hypothalamus/ANS, which are closely linked with the immune system and thereby impact the functional prognosis of RA patients.

The LEE score was associated with several baseline factors, especially higher age and regular use of corticosteroids. Aging and steroid use may contribute to the functioning of emotional responsiveness as assessed using the score, but the present data lacks information that would be helpful for further understanding this issue. However, the association between life events and the ACR class at follow-up in the subgroup with a high LEE score was evident in the model that included age and steroid use as covariates, thus the association would be independent of age and steroid use.

Although antiemotionality was associated with a poor functional prognosis for patients with RA in the present cohort [8], it was not apparent that this behavioral property modified the effects of major life events on the health outcome. Major and minor stressors have been reported to be different in the nature of the immunological response to them by patients with RA [6, 21]. Antiemotionality is a behavioral pattern in response to detected/identified unpleasant emotions. A personality characteristic that overly rationalizes and suppresses sensed feelings would, when such psychological processes are repeated in response to minor daily events, affect the physiological processes, whereas it might have a positive aspect as a coping behavior to a major event, i.e., when strong unpleasant feelings are aroused by the major event, antiemotionality may sometimes be beneficial in realistically dealing with the problems related to the event, which might cancel out the property’s negative aspects.

Several limitations of the study need to be discussed. First, no biomarkers, such as neuroendocrinological or immunological markers were measured, and the underlying pathways that connect emotions to physiological changes are largely unknown. Second, the outcome measure used, ACR class, is limited in its sensitivity in detecting temporal changes of the status [22], and the assessment depends completely on the subjective evaluation of the rheumatologist. Third, a possible recall bias should be considered, because the assessment of life events depended on the subjects’ ability to remember events that happened over the two years before the follow-up. Thus, a person whose emotional responsiveness is diminished may underestimate their life events. Such a bias, however, may not be a major concern because the LEE score was not correlated with the reported number of life events that occurred in the previous year (Spearman’s rank correlation coefficients were −0.04, −0.06, and −0.07 respectively for positive, negative and all life events in the present data). Fourth, the scale used to assess life events was a simple tool that consists of only eight items (see Table 3), thus we could not further discuss the effects of more concrete and more diverse events. The LEE scale is also a simple tool that consists of only four items; it is thought to share its construct to some extent with a component of alexithymia, a personality construct characterized by the sub-clinical inability to identify and describe one’s own emotions [23, 24] which has been linked to physical health problems [25, 26], but this issue has not yet been examined. On the other hand, the simplicity of these tools would simplify replication studies with larger samples or that are done in a daily clinical practice setting. Further attempts to validate the construct that the LEE measures via correlation analyses with relevant psychometrical tools such as the Toronto Alexithymia Scale [27], the Levels of Emotional Awareness Scale [28], and/or with neuroimaging techniques [29] would be useful.

## Conclusions

Major life events, irrespective of their positive or negative nature, were associated with the disease course of patients with RA when the patient has poor ability in identifying emotions as manifest feelings (emotional responsiveness). Thus, emotional responsiveness may be an important contributing factor to the physiological and organic responses that follow major events in the life of patients with RA.

## Abbreviations

ACR: American College of Rheumatology
AISIMS: Assessment and improvement of the system for interdisciplinary medical services for rheumatoid arthritis
ANS: Autonomic nervous system
Antiemotionality: Rational and antiemotional behavior
DMARD: Disease-modifying antirheumatic drug
HPA: Hypothalamus-pituitary-adrenal
RA: Rheumatoid arthritis
RCF: Rationalizing conflicts/frustrations
SI: the Stress inventory
